# Serum calcium, even within normal range, is associated with blood pressure with a potential influence of calcium intake

**DOI:** 10.1210/jendso/bvag031

**Published:** 2026-02-14

**Authors:** Chiara Dal Pont, Andrea Sartorio, Alice Giontella, Elena Rosin, Caterina Cangiano, Valentina Zanconato, Sara Marin, Fabio Bertucci, Silvia Grisenti, Federica Stocchetti, Filippo Cattazzo, Sara Bonafini, Ulrika Ericson, Marju Orho-Melander, Olle Melander, Cristiano Fava

**Affiliations:** Department of Medicine, University of Verona, Verona 371 29, Italy; Department of Medicine, University of Verona, Verona 371 29, Italy; Department of Clinical Sciences, Clinical Research Center, Lund University, Malmö 214 28, Sweden; Department of Medicine, University of Verona, Verona 371 29, Italy; Department of Medicine, University of Verona, Verona 371 29, Italy; Department of Medicine, University of Verona, Verona 371 29, Italy; Department of Medicine, University of Verona, Verona 371 29, Italy; Department of Medicine, University of Verona, Verona 371 29, Italy; Department of Medicine, University of Verona, Verona 371 29, Italy; Department of Medicine, University of Verona, Verona 371 29, Italy; Department of Medicine, University of Verona, Verona 371 29, Italy; Department of Medicine, University of Verona, Verona 371 29, Italy; Department of Clinical Sciences, Clinical Research Center, Lund University, Malmö 214 28, Sweden; Department of Clinical Sciences, Clinical Research Center, Lund University, Malmö 214 28, Sweden; Department of Clinical Sciences, Clinical Research Center, Lund University, Malmö 214 28, Sweden; Department of Emergency and Internal Medicine, Skåne University Hospital, Malmö 214 28, Sweden; Department of Medicine, University of Verona, Verona 371 29, Italy; Department of Clinical Sciences, Clinical Research Center, Lund University, Malmö 214 28, Sweden

**Keywords:** calcium metabolism, blood pressure, hypertension, calcium intake

## Abstract

**Context:**

Even within the physiological range, serum calcium (sCa^++^) may influence vascular tone and blood pressure (BP); however, the potential modulatory role of dietary calcium intake remains unclear.

**Objective:**

To investigate the association between sCa^2+^ and BP (including central BP) and to assess whether calcium intake affects this relationship.

**Methods:**

sCa^2+^ was measured in patients referred for suspected secondary hypertension (VerHyperReg; *n* = 81) and in 2 population-based cohorts: the Malmö Preventive Project (MPP; *n* = 18 240) and the UK Biobank (UKB; *n*≈330 000). Calcium intake was assessed in the Malmö Diet and Cancer Study (MDC; *n* = 28 098) and in a subsample of MPP participants (MPP×MDC; *n* = 4095). Multivariable regression models were used to analyze the associations.

**Results:**

In MPP and UKB, sCa^2+^ was positively associated with systolic and diastolic BP (even within the normal range). In VerHyperReg, sCa^2+^ was associated with central systolic BP. In the MDC, higher calcium intake was inversely associated with BP. In MPP×MDC, sCa^2+^ was positively associated with systolic BP in the lower 3 quartiles of intake, whereas this association was reversed in the highest quartile.

**Conclusion:**

Serum calcium levels within the normal range are positively associated with BP. Increased dietary calcium intake may attenuate this relationship, thus suggesting a modulatory role in BP regulation. Further investigations are warranted.

Hypertension is among the most widespread cardiovascular risk factors and is still the leading cause of death throughout the world [1, 2]. Despite these efforts, it remains largely underdiagnosed and undertreated [3, 4].

Regardless of etiology, the fundamental cardiovascular alteration that leads to hypertension is an increase in systemic vascular resistance [3]. The vascular luminal diameter is regulated by smooth muscle cell contractility, which is controlled by intracellular calcium flux [5].

Cytoplasmic calcium (Ca^++^) signals are responsible for both contractility and relaxation mechanisms in smooth muscle cells, thus resulting in the vasoconstriction and vasodilation of blood vessels, respectively [5, 6].

Calcium homeostasis is important in both muscle contraction and other biological processes (such as cell secretory pathways or cell membrane potential); thus, serum Ca^++^ (sCa^++^) levels and both extracellular and intracellular calcium concentrations are strictly controlled.

Although several studies have documented a positive relationship between sCa^++^ and blood pressure (BP) levels or the presence of hypertension, as well as related comorbidities such as metabolic syndrome and type 2 diabetes [7-9], other studies have not confirmed this association in either the general population or hypertensive patients [10, 11].

Given these conflicting results, we explored several samples (including both large population databases and a cohort of hypertensive patients included in the registry of the European Society of “Hypertension Excellence Center” in Verona) to determine whether a positive correlation exists between BP and sCa^++^ levels (even in the normal range) and to evaluate whether this relationship could be confirmed with both peripheral BP and central BP, as estimated via the SphygmoCor XCEL.

In addition to the previously described link between sCa^++^ and BP, Ca^++^ intake from the diet can influence BP through different mechanisms that are still unclear [12]. For this reason, we evaluated the possible association between Ca^++^ intake and BP, as well as whether an interaction exists between the levels of Ca^++^ intake and sCA^++^ regarding BP levels.

## Materials and methods

Serum Ca^++^ levels were measured after the first clinical evaluation in a group of patients who were referred to our tertiary center known as the European Society of Hypertension (ESH) Hypertension Excellence Center in Verona (*n* = 81); moreover, the patients were included in the Verona Hypertension Register (VerHyperReg; Ethical Committee approval 569CET ASOV, April 2025) because of a suspicion of secondary hypertension, according to the ESH guidelines. Blood samples were analyzed in a single centralized laboratory by using standard methods. In this population, BP was measured as a step involved in the first clinical evaluation by using a validated automated electronic (oscillometric) upper-arm cuff device with the patient in the seated position, in accordance with the ESH guidelines [13]. For each patient, 3 measurements were obtained, and the mean value of the last 2 measurements was considered in our analysis. For these patients, central BP was estimated via tonometry performed with SphygmoCor XCEL (AtCor Medical Pty. Ltd., Sydney, Australia), which is a device that uses brachial and femoral cuff-based acquisition of pulse information to estimate central aortic pressure waveform parameters and carotid-femoral arterial pulse wave velocity [14].

In this study, we also included 2 large population-based studies, including the Malmö Preventive Project (MPP, *n* = 18 240) from Sweden and the UK Biobank (UKB, n∼330 000) from the United Kingdom. The MPP, which is a large preventive case-finding program for cardiovascular risk factors that was launched in 1974, is an urban-based sample composed of middle-aged adults from a town in southern Sweden (approved by the Region Board of Ethics at Lund, number 2009/633) [15]. In this cohort, systolic and diastolic BP (SBP and DBP) were measured twice after 10 minutes of rest in the supine position, and the mean values of BP were then recorded and used in our study [16]. BP values were subsequently adjusted for antihypertensive treatment, with 15 mmHg being added for SBP and 10 mmHg being added for DBP [17]. Blood samples were collected after an overnight fast, and calcium was photometrically measured by using a PRISMA multichannel autoanalyzer (Clinicon AB, Bromma, Sweden) at the Department of Clinical Chemistry, University Hospital, Malmö [18].

The UKB is a large database comprised more than 500 000 middle-aged adults (40-69 years old) from the United Kingdom who were recruited from 2006 to 2010 [19, 20]. Individual data were accessed by using application ID 76658 in compliance with the UKB Ethics Advisory Committee (https://www.ukbiobank.ac.uk/ethics/) and with the Swedish Ethical Review Authority (approval number: 2022-01085-01). Blood pressure was obtained in this population as a step involved in the first assessment. Blood pressure was measured twice by using a validated automated electronic (oscillometric) upper-arm cuff device with the patients in the seated position and after 5 minutes of seated rest [21]. The mean values of the 2 measurements were calculated; moreover, values of 15 and 10 mmHg were added to the SBP and DBP measurements, respectively, in the case of antihypertensive treatment [17]. Blood samples were collected during the recruitment visit.

Ca^++^ intake was available in another urban-based cohort from Malmö known as the Malmö Diet and Cancer (MDC, *n* = 28 098) cohort [22]. The study protocol was approved by the Ethics Committee at the Medical Faculty at Lund University (approval number: LU 51/90). In this sample, BP was measured in the seated position after 5 minutes of rest [23]. As in the other cohorts, BP values were adjusted for antihypertensive medication [17]. Estimated milligrams per day of Ca^++^ intake were assessed for each individual by combining information from the following 3 methods: a 7-day food diary (“Menybok”), a 168-item semiquantitative diet history questionnaire (“Kosthistoria”) and a 45-60 minutes diet history interview [24]. An approach combining food record and quantitative food frequency questionnaire was shown to be more effective in capturing overall dietary patterns than an extensive food frequency questionnaire alone [25, 26]. Information from the different sources was summarized as the average daily consumption, as previously described [24]. The values of Ca^++^ intake were adjusted for the estimated daily total energy intake and expressed as Megajoules.

For 4095 participants of the MPP who also subsequently participated in the MDC study (hereafter referred to as the MPP×MDC), we obtained data on both sCa^++^ and Ca^++^ intake. We used this subsample to evaluate whether the association between sCa^++^ was influenced by the interaction with Ca^++^ intake. For this aim, we divided the cohorts into quartiles of Ca^++^ intake and compared the association between sCa^++^ and BP across the 4 quartiles.

Statistical analysis was conducted by using Jamovi (ver. 2.3.18) and The Jamovi project (2022 [Computer Software]; retrieved from https://www.jamovi.org). Continuous variables are reported as the means and SDs. Categorical variables are expressed as percentages and absolute numbers. Linear regression models adjusted for well-known potential confounders (that are age, sex, and body mass index [BMI]) were used to assess the association between sCa^++^ or dietary Ca^++^ intake and BP values. The results of the linear regression are presented as beta values (95% CIs). A sensitivity analysis was performed to verify whether the association between sCa^++^ and BP persisted after excluding individuals treated with diuretics. A *P* value <.05 was considered to indicate statistical significance.

## Results

The demographic, anthropometric, and BP data of the 5 samples (VerHyperReg, MPP, MDC, MPP×MDC and UKB) are presented in Table 1. Ages across the cohorts ranged from 45.2 ± 7.4 (MPP) to 58.1 ± 7.6 (MDC) years. VerHyperReg included a greater percentage of males compared to the other cohorts; moreover, with respect to BMI, participants in VerHyperReg, MDC and the UKB exhibited mean values greater than the overweight threshold (25 kg/m^2^). The mean SBP values ranged from 128 ± 15.5 to 153.2 ± 18.7 mmHg, with VerHyperReg, MDC and UKB slightly exceeding the hypertension threshold (140 mmHg). DBP values were quite similar across the cohorts.

**Table 1:** General characteristics of the different populations included in the present study

Variables	VerHyperReg (*n* = 81)	MPP (*n* = 18 240)	MDC (*n* = 28 098)	UKB (*n* = 330 000)	MPP×MDC (*n* = 4095)
Age, years	46 ± 13.5	45.2 ± 7.4	58.1 ± 7.6	57.2 ± 8.1	46.8 ± 6.29
Sex, % (male)	60.5	36.6	56.2	45.9	55
BMI, kg/m^2^	25.8 ± 3.5	24.3 ± 3.4	25.4 ± 4.0	27.5 ± 4.8	24.3 ± 3.3
SBP, mmHg	143 ± 18.1	128.1 ± 15.4	141.8 ± 20.3	153.2 ± 18.7	128 ± 15.5
DBP, mmHg	87.6 ± 13.2	84.5 ± 9.5	89.4 ± 17.3	92.2 ± 10.2	84.5 ± 9.44
Central SBP, mmHg	131 ± 14.9	n.a.	n.a.	n.a.	n.a.
Central DBP, mmHg	89.9 ± 11.4	n.a.	n.a.	n.a.	n.a.
sCa^++^, mmol/L	2.37 ± 0.08	2.39 ± 0.09	n.a.	2.38 ± 0.09	2.38 ± 0.09
Ca^++^ intake, mg/day	n.a.	n.a.	1126.3 ± 413.38	n.a.	1102.0 ± 389.0

VerHyperReg, Verona Hypertension Register; MPP, Malmö Preventive Project; MDC, Malmö Diet and Cancer; UKB, UK Biobank; MPP×MDC, participants of the MPP who were successively examined in the MDC; BMI, body mass index; Ca^++^ intake, daily calcium intake; DBP, diastolic blood pressure; SCa^++^, serum calcium level; SBP, systolic blood pressure; n.a., not available.

The associations between sCa^++^ and BP across all of the cohorts, as well as between Ca^++^ intake and BP for MPP×MDC, are presented in Table 2.

**Table 2:** Linear associations of sCa^++^ and Ca^++^ intake with systolic and diastolic BP in the different samples

		Systolic BP, mmHg*^a^*	Diastolic BP, mmHg*^a^*
		β (95% CI)	β (95% CI)
**VerHyperReg**	**sCa^++^**	0.202 (−0.02; 0.425)	0.023 (−0.211; 0.258)
**VerHyperReg**	**sCa^++^**	Central systolic BP 0.229* (0.013; 0.445)	Central diastolic BP 0.13 (−0.087; 0.348)
**MPP**	**sCa^++^**	1.56*** (1.34; 1.78)	0.93*** (0.80; 1.07)
**UKB**	**sCa^++^**	1.79*** (1.74; 1.85)	0.98*** (0.95; 1.02)
**MPP×MDC**	**sCa^++^**	0.08*** (0.05; 0.11)	0.063*** (0.033; 0.094)
**MDC**	**Ca^++^** **intake**	−0.028*** (−0.039; −0.017)	−0.032*** (−0.044; −0.021)
**MPP×MDC**	**Ca^++^** **intake**	0.007 (−0.022; 0.036)	0.008 (−0.022; 0.037)

The reported associations refer to peripheral systolic and diastolic BP, unless otherwise specified. The results refer to linear regression models adjusted for sex, age, and body mass index.

VerHyperReg, Verona Hypertension Register; MPP, Malmö Preventive Project; mmHg, millimeters of mercury; MDC, Malmö Diet and Cancer; UKB, UK Biobank; MPP×MDC, participants of the MPP who were successively examined in the MDC; CI, confidence interval; BP, blood pressure.

^a^millimeters of mercury.

^*^: *P* < .05; ***: *P* < .001.

In VerHyperReg, sCa^++^ was associated with central BP after adjustments for age, sex and BMI. With respect to the MPP, sCa^++^ was associated with both SBP (+1.56 [1.34-1.78]; *P* < .001) and DBP (+0.93 [0.80-1.07]; *P* < .001) after adjustments for age, sex, and BMI. The association was also maintained in a sensitivity analysis after excluding individuals who received diuretic treatments (*n* = 15 513; Table 3). Similarly, in the UKB, a significant association between both SBP (+1.79 [1.74-1.85]; *P* < .001) and DBP (+0.98 [0.95-1.02]; *P* < .001) and sCa^++^ was observed after adjustments for the same covariates (Table 2).

**Table 3:** Sensitivity analysis: association of sCa^++^ with BP after excluding individuals under diuretic treatment in the MPP cohort (*n* = 15 513)

		Systolic BP, mmHg*^a^*	Diastolic BP, mmHg*^a^*
		β (95% CI)	β (95% CI)
**MPP**	**sCa^++^**	1.45*** (1.22; 1.67)	0.86*** (0.73; 1.00)

The results refer to linear regression models adjusted for sex, age and body mass index. BP, blood pressure; CI, confidence interval; MPP, Malmö Preventive Project; mmHg, millimeters of mercury.

^*a*^millimeters of mercury. ***: *P* < .001.

As shown in the same table, a significant and inverse association between daily Ca^++^ intake and both SBP (−0.03 [−0.04, −0.02]; *P* < .001) and DBP (−0.03 [−0.04, −0.02]; *P* < .001) was observed in the MDC. In the MPP×MDC subsample, when the participants were divided into quartiles according to Ca^++^ intake, a significant and direct association of sCa^++^ with SBP was observed in the first 3 quartiles of Ca^++^ intake (+1.75 [1.08/1.87], *P* < .001; +1.01 [0.31/2.00], *P* = .04; +0.97 [0.51/1.90], *P* = .04, respectively), whereas in the fourth quartile, an inverse association was observed (−0.97 [−1.85/−0.10]; *P* = .03), although it was demonstrated to be statistically significant (Table 4).

**Table 4:** Association between sCa^++^ and blood pressure in participants in different quartiles of Ca^++^ intake in the MPP×MDC intersection subsample (*n* = 4095) evaluated via linear regression

			Systolic BP, mmHg*^a^*		Diastolic BP, mmHg		
		β	95% CI	*P*	β	95% CI	*P*
Ca^++^ intake (MJ adjusted)	Q1	1.75	1.08/1.87	<.001	0.35	−0.22/0.90	.22
	Q2	1.01	0.31/2.00	.04	0.73	0.16/1.30	.01
	Q3	0.97	0.51/1.90	.039	0.44	−0.16/1.04	.15
	Q4	−0.97	−1.85/−0.10	.03	−0.01	−0.53/0.51	.96
sCa^++^ (SD unit)							

The results refer to linear regression models adjusted for sex, age, and body mass index.

MPP×MDC, participants of the MPP who were successively included in the MDC; CI, confidence interval; BP, blood pressure; Ca^++^, calcium intake; mmHg, millimetersof mercury; MJ, megajoule; Q, quartile of calcium intake; sCa^++^, serum calcium level.

^*a*^millimeters of mercury.

## Discussion

The current study primarily demonstrated that sCa^++^ (even in the normal range) is consistently associated with both peripheral and central BP; moreover, this association is influenced by Ca^++^ intake, as evidenced by the stratified analysis.

Although the effect of serum calcium levels on BP has been investigated for at least 30 years, previous studies have reported conflicting results. Interestingly, a large cross-sectional study involving 26 778 US adults from the 2007-2018 National Health and Nutrition Examination Survey (NHANES) revealed a positive association between higher sCa^++^ concentrations and the incidence of hypertension; however, this was only observed at an initial concentration of 9.4 mg/dL [7], thus confirming a previous observation in a more limited sample of 12 405 US adults from the third NHANES [27]. Similarly, a recent longitudinal study revealed a positive association between serum calcium levels and increased prevalence of hypertension, diabetes, and metabolic syndrome [28]. Moreover, in another study conducted by Patel et al [11], among 9260 hypertensive adults, sCa^++^ significantly predicted all-cause and noncardiovascular mortality in both men and women.

Using a Mendelian randomization study, our group recently reported that genetically determined serum calcium levels (but not parathyroid hormone [PTH] or vitamin D levels) are causally associated with BP levels [29], thus further supporting the pivotal role of this vital cation in BP.

Conversely, other studies (including large population-based studies) have not determined any association between serum calcium levels and hypertension [10, 11, 30-33].

Despite the extensive research on this topic, the possible mechanisms that control serum calcium levels and the proposed effects on BP are not fully understood. Intracellular and extracellular Ca^++^ concentrations are fundamental for regulating the contractility of vascular smooth muscle cells, endothelial cell function, and cell proliferation. The dysregulation of Ca^++^ signaling has been implicated in vascular smooth cell hyperactivity and vascular remodeling, both of which are characteristic features of hypertension pathogenesis [34]. Moreover, Ca^++^ sensing receptors, which are mainly expressed by parathyroid glands, renal tubules and the brain, as well as by adipocytes, are key structures in detecting extracellular Ca^++^ variations [8, 35]. Furthermore, calcium-regulating hormones, particularly PTH, as well as vitamin D and fibroblast growth factor 23 (FGF23), can directly affect BP, in addition to altering sCa^++^ levels, although the detailed pathophysiological mechanisms remain unclear [9, 29, 36-38].

In our study, we considered 3 large population-based databases, including the MPP (*n* = 18 240), MDC (*n* = 28 098), and the UKB (*n*∼330 000). The MPP and the MDC can be considered to be mostly urban-based samples composed of middle-aged adults from a town in southern Sweden [15]. The UKB is a large database founded in the United Kingdom, which involves 500 000 adults throughout the country [20]. We also separately analyzed a subsample of 4095 MPP participants who also participated in the MDC (known as the MPP ×MDC) [22]. Interestingly, a direct association between sCa^++^ and BP was observed in all of the investigated samples after adjustments for sex, age, and BMI; however, notable differences in the beta values were detected among the samples. This result may be explained by differences in age between the analyzed populations. Our results confirm and reinforce the findings from other studies [7, 25, 26], although several differences were observed between these studies and our study. For example, we included a larger number of participants from multiple European cohort studies (including the MDC, MPP, and UKB) characterized by considerable heterogeneity in terms of demographic and clinical backgrounds, thereby providing a more inclusive representation of the general population. Moreover, these findings differ considerably from those of Patel et al and VerHyperReg, which exclusively focused on hypertensive patients [11]; additionally, the findings more closely align with those of the NANHES studies, which included many adults in the United States [7, 25]. The average level of sCa++ was observed to be quite similar at approximately 2.3-2.4 mmol/L in all of these studies, whereas the average BP values were considerably lower in the MPP.

In addition to large cohort studies, we analyzed a sample of patients from the ESH Excellence Center in Verona (VerHyperReg). This sample included hypertensive patients who were referred to our center for suspected secondary forms of hypertension. For this cohort of patients, we were able to estimate central BP rather than peripheral BP. Due to the amplification of sphygmic waves and other hemodynamic phenomena, peripheral BP is not as strongly associated with organ damage as central BP is, as we and others have reported in previous studies analyzing diabetes [39, 40] and hypertension [41]. Thus, central BP can be a more accurate measure of actual aortic pressure in direct association with visceral artery hypertension-mediated target organ damage.

Notably, we observed a positive association between sCa^++^ and central SBP after adjustments for sex, age, and BMI (even if the effect size of the association was modest). Nevertheless, to our knowledge, this is the first study to evaluate the impact of sCa^++^ levels on central BP.

Some studies have evaluated how calcium intake can influence sCa^++^ levels. In particular, human and animal data have demonstrated that low calcium intake is associated with high BP values [12]. Indeed, Ca^++^ intake may modify intracellular Ca^++^ levels in vascular smooth muscle cells, and it is involved in vascular volume variation through the renin–angiotensin–aldosterone system. Another notable link is observed between hypertension in rats and higher levels of PTH and calcitriol due to low calcium intake [42]. Moreover, several studies (including randomized clinical trials) have highlighted the association between low calcium intake and both pregnancy-induced hypertension and a reduction in BP after calcium supplementation [43, 44].

We assessed calcium intake in 2 related samples, including the entire MDC and the subgroup MPP×MDC. In the MDC, we observed a negative association between calcium intake (which was evaluated via a detailed multi-item questionnaire) and both SBP and DBP.

In the MPP×MDC sample, even though we did not identify a significant association in the linear regression for the whole sample, after stratification for the quantity of Ca^++^ intake (as shown in Table 4), we observed that for the first 3 quartiles, a direct association between serum calcium and SBP was present; however, in the last quartile, an inverse association was demonstrated. The observation of the inverse association between serum calcium concentration and SBP in the last quartile is particularly interesting because calcium intake can modulate the effect of serum calcium concentration on BP, thereby suggesting a possible “therapeutic” effect. To our knowledge, this study is the first to include an analysis of the interaction of calcium intake, thus representing a novelty in the field.

### Limitations

This study has several limitations. First, regarding the UKB and MPP, peripheral BP values were measured during a single visit; additionally, only a single measurement was often available, with possible detection bias being present. Moreover, due to the vast number of samples in these cohorts, our analysis did not consider the possible impacts of some important factors, such as the different features of the 2 populations, comorbidities, or the presence or absence of antihypertensive medications. With respect to the Verona ESH Center, the small sample size can indicate a weakness of our study, even if the measurement of central BP provides important information. Furthermore, in our study, we considered serum calcium levels and not ionized serum calcium levels. Furthermore, the estimation of calcium intake from questionnaires (even if these questionnaires are extremely detailed and implemented via interviews) is subject to approximations, and the specific source of calcium (ie, from dairy products or vegetables) is difficult to differentiate. In addition, the role of hormones (such as PTH or vitamin D) in the relationship between BP and both serum calcium and calcium intake was not considered in our analysis, due to the fact that we did not have information concerning their dosage. Conversely, our previous Mendelian randomization study revealed a primary role for these hormones in BP homeostasis. Finally, the observational designs of our studies cannot support a cause–effect interpretation of the results.

## Future perspectives

The results of the present study confirm the presence of a direct (although modest) correlation between BP and serum calcium levels (even in the normal range), which align with the findings of other previous studies and demonstrate that calcium intake can mitigate this positive association and possibly reverse it. Thus, a higher intake of Ca^++^ may counterbalance the deleterious effect of high sCa^++^ on BP.

Further studies are needed to confirm our results and to examine the role of the hormones that are involved in calcium homeostasis in this interaction and to better define the molecular pathways involved in this association.

## Data Availability

Some or all of the datasets generated during and/or analyzed during the current study are not publicly available; however, they are available from the corresponding author upon reasonable request.

## Abbreviations

BMI: body mass index
BP: blood pressure
ESH: European Society of Hypertension
MDC: Malmö Diet and Cancer Study
MPP: Malmö Preventive Project
NHANES: National Health and Nutrition Examination Survey
PTH: parathyroid hormone
UKB: UK Biobank
